# A TaqMan Real-Time PCR Assay for Detection and Quantification of *Sporisorium scitamineum* in Sugarcane

**DOI:** 10.1155/2013/942682

**Published:** 2013-10-21

**Authors:** Yachun Su, Shanshan Wang, Jinlong Guo, Bantong Xue, Liping Xu, Youxiong Que

**Affiliations:** Key Laboratory of Sugarcane Biology and Genetic Breeding, Ministry of Agriculture/Fujian Agriculture and Forestry University, Fuzhou 350002, China

## Abstract

*Sporisorium scitamineum* is a fungal smut pathogen epidemic in sugarcane producing areas. Early detection and proper identification of the smut are an essential requirement in its management practice. In this study, we developed a TaqMan real-time PCR assay using specific primers (bEQ-F/bEQ-R) and a TaqMan probe (bEQ-P) which were designed based on the *bE* (b East mating type) gene (Genbank Accession no. U61290.1). This method was more sensitive (a detection limit of 10 ag pbE DNA and 0.8 ng sugarcane genomic DNA) than that of conventional PCR (10 fg and 100 ng, resp.). Reliability was demonstrated through the positive detection of samples collected from artificially inoculated sugarcane plantlets (FN40). This assay was capable of detecting the smut pathogen at the initial stage (12 h) of infection and suitable for inspection of sugarcane pathogen-free seed cane and seedlings. Furthermore, quantification of pathogen was verified in pathogen-challenged buds in different sugarcane genotypes, which suggested its feasibility for evaluation of smut resistance in different sugarcane genotypes. Taken together, this novel assay can be used as a diagnostic tool for sensitive, accurate, fast, and quantitative detection of the smut pathogen especially for asymptomatic seed cane or plants and evaluation of smut resistance of sugarcane genotypes.

## 1. Introduction

Sugarcane smut (*Sporisorium scitamineum*) is a disease worldwide and presents in almost all sugarcane-planting countries [1, 2]. The typical feature of sugarcane infected with the smut is the emergence of black whips around 120 days of planting [3]. It is impossible to completely eliminate this disease once the smut appears, due to the enormous quantity of teliospores and the quick spread within the sugarcane growing area. The infected buds may either produce symptoms or exist as a latent infection which may germinate and produce black whips in the following season [4]. The smut spores germinate on the internal surface followed by formation of appressoria on the inner scales of the young buds as well as the base of the emerging leaves. Entry into the meristem of the buds occurs between 6 h and 36 h after the teliospore deposition [5]. The disease commonly produces plants with profuse tillering, narrow leaves, spindly shoots, and poor cane, therefore causing serious losses in cane yield and sugar yield per unit area.

 The development and severity of the smut disease depend on environmental conditions, the resistance of sugarcane genotypes, and the interaction between sugarcane, *S. sporisorium,* and environment. Previous researches revealed that the control of sugarcane smut is difficult and the successful management relies mainly on the use of resistant cultivars or pathogen-free seed cane and quarantine [3, 6]. Conventional diagnostic approaches involve the application of morphological identification, which is time consuming and thus not conducive to control the spread of disease for the three months of disease period [3], or the isolation of the pathogen followed by biochemical identification and pathogenicity tests (requires more than one week) [7], or serological testing which requires high quality antibodies [8], or electron microscopy which needs expensive equipment [8]. Until now, none of sugarcane smut genome information has been reported yet. Albert and Schenck [9] had successfully amplified smut with the bE4/bE8 primers based on the *Ustilago maydis bE* mating-type gene, and its specific conventional PCR product (459 bp) (Genbank Accession no. U61290.1) was found to be approximately 70% identical in nucleotide sequence to the corresponding region of the *bE* gene in *Ustilago maydis* and *Ustilago hordei*. Southern analysis of *S. scitamineum* revealed a specific hybridization with smut DNA rather than with uninfected sugarcane DNA or with DNA from *Arthrobotrys musiformis*. PCR amplification from the *bE* gene was used as an assay for the presence of smut DNA, and it was also validated by several other scientists [3, 9]. However, one of the limitations of the conventional PCR method is that they do not allow quantifying the amount of *S. scitamineum* in the sugarcane, and there is no report on sensitive detection and quantification assay for this pathogen.

 The exact detection and proper quantification of the smut is therefore an essential requirement for an effective management practice. Establishing a sensitive TaqMan real-time PCR detection method would be useful for quarantine, epidemiological, research, and smut control. This could be applied to (i) sugarcane seeds or stalks importation and export inspection for smut pathogen, (ii) evaluation of smut resistance between or within several or a batch of sugarcane genotypes by quantifying the copy number of smut pathogen in asymptomatic smut-infected sugarcane, and (iii) supervision and management of the pathogen-free seed cane of sugarcane.

 In the present study, a set of TaqMan real-time PCR primers and a probe, which were reliable and specific, were designed according to the sequences of smut *bE* gene and used for the detection and quantification of smut in sugarcane. Here, for the first time, we report the development of a TaqMan real-time PCR assay for the smut, which is compared with conventional PCR, is much more sensitive. Based on this system, the above three expected objectives were achieved. Furthermore, the teliospore germination process and the curve of mycelial growth were observed in order to get a better determination on the key timing of sugarcane-smut interaction.

## 2. Materials and Methods

### 2.1. Fungal Isolates and Growth Conditions

 Strain of sugarcane-smut pathogen *S. scitamineum* was selected for the development of TaqMan real-time PCR assay. Smut-infected whips were collected from sugarcane cultivar “ROC”22 cultivated in the Key Laboratory of Sugarcane Biology and Genetic Breeding, Ministry of Agriculture (Fuzhou, China). Teliospores were mixed and sealed in plastic bags and then stored at 4°C. This was used for gradient dilution in sterile water and plated onto potato dextrose agar (PDA) containing 75 *μ*g/mL streptomycin (Shenggong, China). Plates were incubated in the dark for 4 d at 28°C. Single colonies were transferred onto the new PDA medium and cultured at 28°C for 5 d. Fungal mycelial DNA was extracted using the SDS method [10] and eluted with sterile water containing 100 *μ*g/mL RNase A. The DNA sample was quantified using the absorbance at 260 nm, 280 nm by NanoVue plus (GE, USA) and stored at −20°C for later use.

### 2.2. Primers and Probe Design and Specificity Test

 The sequence of the smut *bE *gene was chosen as the PCR target gene for confirmation of the presence of smut, as reported by Albert and Schenck [9]. PCR amplification was performed in a total volume of 25 *μ*L containing 100 ng of smut DNA, 400 nM each of the primers *b*E4 (5′-CGCTCTGGTTCATCAACG-3′) and *b*E8 (5′-TGCTGTCGATGGAAGGTGT-3′), 2.5 *μ*L 10 × *Ex Taq* Buffer (Mg^2+^ Plus), 200 *μ*M dNTPs, and 0.625 U *Ex Taq* DNA polymerase (TaKaRa, China). PCR comprised the following steps: 94°C, 4 min; 94°C, 55°C, and 72°C, 30 s each, 35 cycles; 72°C, 10 min [9]. The amplified *bE* fragment (459 bp) was purified from a 1.5% agarose gel and cloned into *Escherichia coli* DH5*α* cell, using pMD18-T vector (TaKaRa, China). One positive clone was referred to as pbE. The DNA of the recombinant plasmid was extracted using Plasmid Mini Kit I (OMEGA, China) and sent for sequencing (Shenggong, China) in order to ensure that the target DNA sequence was amplified.

 The TaqMan probe (bEQ-P) and the primers (bEQ-F/bEQ-R) were designed according to the sequence of smut *bE *gene using the Primer Express software version 2.0 (ABI Applied Biosystems, USA). Primers bEQ-F (5′-TGAAAGTTCTCATGCAAGCC-3′) and bEQ-R (5′-TGAGAGGTCGATTGAGGTTG-3′) were designed to yield a 123 bp fragment of the *bE* gene. The TaqMan probed bEQ-P (5′-TGCTCGACGCCAATTCGGAG-3′) contained 6-carboxy-fluorescein (FAM) reporter dye at the 5′ end and 6-carboxytetramethylrhodamine (TAMRA) fluorescent quencher at the 3′ end. The sequences used to design testing primers and probe were compared to other organisms in National Center for Biotechnology Information (NCBI, http://www.ncbi.nlm.nih.gov/) to confirm the specificity and were commercially synthesized by TaKaRa Biotechnology (Dalian, China).

The specificity of PCR-based TaqMan assay was performed using 100 ng DNA of *S. scitamineum*, *Phoma* sp., *Fusarium moniliforme*,* Pestalotia ginkgo*, *Fusarium oxysporum*, and *Helminthosporium sacchari*. Meanwhile, cane exudates from sugarcane genotypes Yacheng05-179 (smut resistance) (private bulletin) and “ROC”22 (smut susceptible) (private bulletin) were cultivated at PDA liquid medium containing 75 *μ*g/mL streptomycin at 200 rpm at 28°C overnight in order to obtain the endophytes, respectively. All eight tested fungal DNA preparations were extracted by SDS method.

### 2.3. TaqMan Real-Time PCR Conditions and Standard Curves

TaqMan real-time PCR was performed in an ABI 7500 real-time PCR system (Applied Biosystems, USA). Amplification reaction contained 12.5 *μ*L of 2 × TaqMan Universal Master Mix (Roche, Shanghai, China), 1.0 *μ*L each of 10 *μ*M primers (bEQ-F and bEQ-R), 0.2 *μ*L of 10 *μ*M probe, and 1.0 *μ*L of template DNA and nuclease-free water to a final volume of 25 *μ*L. The thermal profile consisted of 50°C for 2 min, followed by 95°C for 10 min and 40 cycles of 95°C for 15 s and 60°C for 1 min. Fluorescence was measured once per cycle at the end of the 60°C segment.

 The standard curve was generated using tenfold serial dilutions with ddH_2_O from 10^−3^ to 10^−10^ (approximately 1.987 × 10^8^ ~ 1.987 × 10^1^ copies/*μ*L) of 100 ng/*μ*L pbE DNA. The PCR conditions for this standard curve were adopted to perform the further reactions to estimate the copy number of smut pathogen by TaqMan real-time PCR assay.

### 2.4. Sensitivity Comparisons of TaqMan Real-Time PCR and Conventional PCR

The sensitivity for the detection and quantification limits of the primers was investigated based on the plasmid with a known copy number of the *bE* insert. The pbE DNA (100 ng/*μ*L) was tenfold serial diluted, 10^−3^ to 10^−10^. Then dilutions were detected by TaqMan real-time PCR as described above. The gene copy number was calculated as follows: Copies/mL = 6.02 × 10^23^  × (concentration g/mL)/(MW g/mol); MW = genome length × 660 dalton/bp [11]. In parallel, the same sample was detected by conventional PCR method [9]. Conventional PCR amplified product with a length of 459 bp was detected on 1.5% agarose gel. The end-point dilution of these two assays in which a positive result was recorded was compared.

In order to further evaluate the detection accuracy and sensitivity in sugarcane, three pieces of the youngest fully expanded leaf, namely, +1 leaf, which with a visible dewlap (the collar between the leaf blade and sheath), were collected from smut-infected plants of 10 months old of cultivar “ROC”22 and used for detection analysis by both real-time PCR and conventional PCR assay. Two microlitres of fivefold serial dilutions (500, 100, 20, 4.0, and 0.8 ng/*μ*L) of +1 leaf DNA was used as templates, and TaqMan and conventional PCR assay were performed in the protocols as described above.

### 2.5. Germination Process and Growth Curve of Smut Teliospores

As for understanding the biological characteristics of *S. sporisorium*, the germination process and the growth curve of smut teliospores were observed. By germination assay, teliospores from smut whip were transferred onto the PDA liquid medium containing 75 *μ*g/mL streptomycin and cultured at 140 rpm at 28°C. The teliospore germination periods were then monitored by microscopy (Axio Scope A1, Germany) at 0 h, 6 h, 12 h, 24 h, 36 h, and 48 h after cultivation [12]. For growth curve observation, smut spores were eluted to 5 × 10^6^ spores/mL with 300 mL sterile PDA liquid medium (containing 75 *μ*g/mL streptomycin) and cultured at 140 rpm at 28°C [10]. During the culture process, a spectrophotometer (Lambda35; Perkin Elmer, America) was used to determine the optical density at 600 nm at 25 time points at set intervals between 0 h and 288 h. The growth curves were drawn by OriginPro 8.0 software with culture time as abscissa and OD_600_ value as ordinate.

### 2.6. Detection and Quantification of *S. sporisorium* in Sugarcane Plantlets Inoculated with Smut

Sugarcane cultivar FN40 (a widely grown cultivar in China) was provided by the Key Laboratory of Sugarcane Biology and Genetic Breeding, Ministry of Agriculture (Fuzhou, China). The pathogen-free FN40 four-month-old plantlets were syringe-inoculated from the basal portion up to 2 cm length with 0.5 *μ*L of the smut suspension containing 5 × 10^6^ spores/mL in 0.01% (v/v) Tween-20 [7, 13]. All plantlets were incubated at 28°C under conditions of 12 h light and 12 h darkness. At each time point of 0 h, 12 h, 24 h, 48 h, 120 h, 168 h, and 336 h after inoculation treatment, three culms were sampled for DNA extraction by CTAB-based protocol as reported [14]. These DNA samples were subjected to the TaqMan assay.

### 2.7. Smut Resistance Evaluation Using TaqMan Real-Time PCR

Two-bud sets of the sugarcane genotypes of Yacheng05-179 (resistant) and “ROC”22 (susceptible) were inoculated with 0.5 *μ*L smut suspension as described above. After that, five buds from both varieties were excised at 0 h, 12 h, 24 h, 48 h, and 168 h after inoculation, respectively. Collected samples were washed with distilled water, frozen in liquid nitrogen, and stored at −80°C until extraction of DNA. Quantification of smut pathogen in the buds was accomplished by calculating the target amplicon copy number based on TaqMan real-time PCR.

### 2.8. Statistical Analysis

Each run of TaqMan real-time PCR contained three replicates, as well as three smut-DNA-template positive controls, mock controls (pathogen-free plantlets syringe-inoculated with sterile ddH_2_O), and blank controls (without template DNA). For the evaluation of TaqMan assay efficiency and the calculation of the copy number of the smut pathogen, a standard curve was generated using pbE DNA in each TaqMan assay. Data analyses were performed using Microsoft Excel and OriginPro 8.0 software. Standard curve analysis was based on threshold cycles values (Ct) and serial dilutions of pbE DNA (10^−3^ to 10^−10^). The Δ*R*_*n*_ (change in normalised fluorescence) records the amount of the product amplified. Correlation coefficient (*R* ^2^), slope (*S*), and efficiency (*E*(%) = 10^(1/*S*)^ − 1) [15, 16] of amplification were calculated to assess the linear range and reliability of the TaqMan real-time PCR assay. By the equation of the linear regression line (*y* = *ax* + *b*) (*a* was the slope and *b* was the intercept), the copy number can be calculated in sugarcane samples [17]. The formula for the copy number was inferred from the equation: copy number = *λ* × 10^(Ct−*b*)/*a*^ [18]. In this study, *λ* was a fixed value as 1.987 × 10^11^ for the dynamic range of smut which was obtained by the copy number for each serial dilution (100 pg → 10 ag). The Ct value below 35 indicated a positive result [19]. If the Ct value exceeds 35, the sample should be detected again, from which no Ct value means negative, otherwise positive.

## 3. Results

### 3.1. Design of Primers and Probe

 A BLAST comparison of the 123 bp region of the smut *bE* gene, for which the primers were designed, showed no similarity with other sequences but had a 100% sequence identity with the target *bE* gene in GenBank. Furthermore, using BLAST, the sequences of the primers and probe did not completely match any sequences of other published organisms. In all TaqMan real-time PCR reactions, no fluorescence was observed for the negative (mock and blank) samples, but successful detection was observed in positive controls (pbE and smut DNA). In addition, species specificity was evaluated by testing DNA of some common encountered fungal diseases on sugarcane (*Phoma* sp., *Fusarium moniliforme*,* Pestalotia ginkgo*, *Fusarium oxysporum*, and *Helminthosporium sacchari*) and endophytes of Yacheng05-179 and “ROC”22, by TaqMan assay under the same condition. No positive result was observed in these samples. Therefore, the primers bEQ-F/bEQ-R and probe bEQ-P were selected for further experimentation.

### 3.2. Standard Curves and Amplification Efficiency

**Figure 1:**
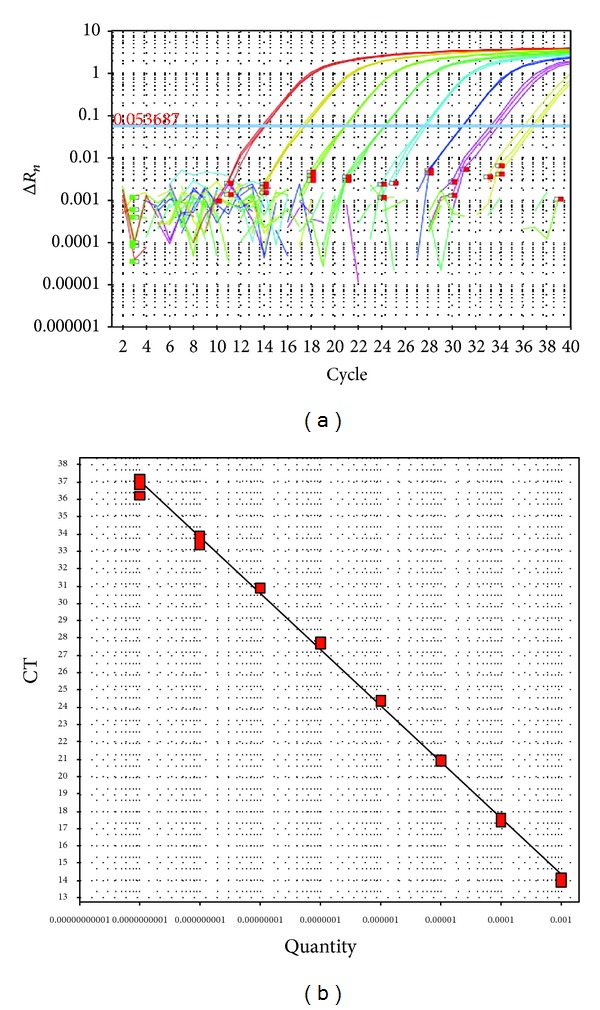
Sensitivity test of TaqMan real-time PCR assay for ten fold serial dilutions of smut pbE DNA standard. (a) The amplification plots showed the testing results of a ten fold dilution serials containing the following pbE DNA: 100 pg, 10 pg, 1 pg, 100 fg, 10 fg, 1 fg, 100 ag, and 10 ag, while no amplification signals were observed in the mock and blank controls. (b) The linear regression analyses between the quantities of templates and Ct values. Regression equations were calculated with *y* = − 3.247*x* + 4.629, *R* ^2^ = 0.998, *S* = − 3.247, and *E*(%) = 1.032.

In generated standard curves, linearity between the TaqMan real-time PCR Ct values and target concentration was observed over eight orders of magnitude in ten-fold serial dilutions in triplicate (Figure 1). Initial quantities of smut pbE DNA templates were 100 pg, 10 pg, 1 pg, 100 fg, 10 fg, 1 fg, 100 ag, and 10 ag, approximately 1.987 × 10^8^ to 1.987 × 10^1^ copies, respectively. Ct value ranged from 14 to 37 (Figure 1(a)). A linear regression analysis (*R* ^2^ = 0.998; *E*(%) = 1.032), which revealed a linear relationship between the quantities of templates and Ct values, was performed with Δ*R*_*n*_ and Ct, and the results were shown in Figure 1(b), which demonstrated that the TaqMan real-time PCR protocol was feasible to smut pathogen quantification.

### 3.3. Comparison of the Sensitivity of TaqMan Real-Time PCR Assay and Conventional PCR

**Figure 2:**
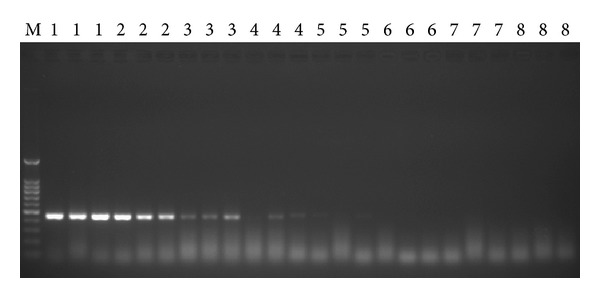
Sensitivity test of conventional PCR based on eight ten-fold serial dilutions of smut pbE DNA. M: 100 bp DNA ladder marker; Lane 1, 100 pg/*μ*L; Lane 2, 10 pg/*μ*L; Lane 3, 1 pg/*μ*L; Lane 4, 100 fg/*μ*L; Lane 5, 10 fg/*μ*L; Lane 6, 1 fg/*μ*L; Lane 7, 100 ag/*μ*L; Lane 8, 10 ag/*μ*L.

Eight ten-fold serial dilutions of pbE DNA were assayed to determine the detection sensitivity by two different methods. This assay showed excellent results with regard to sensitivity, compared to conventional PCR. Stable amplification was observed for as low as 10 ag of pbE DNA in three replications of TaqMan real-time PCR assay (Figure 1), indicating the lowest limit of smut detection by this assay was 1.987 × 10^1^ copies. In conventional PCR, the expected amplicon of 459 bp was obtained in the reaction by detection on agarose gel and sequenced, and the detection limit was 10 fg which is equivalent to 1.987 × 10^4^ copies (Figure 2). The above results demonstrated that the application of TaqMan real-time PCR assay in relatively accurate quantification of the target DNA was possible, which also showed a wider dynamic range of nearly 1000 (1.987 × 10^4^/1.987 × 10^1^) times more sensitivity than conventional PCR.

**Figure 3:**
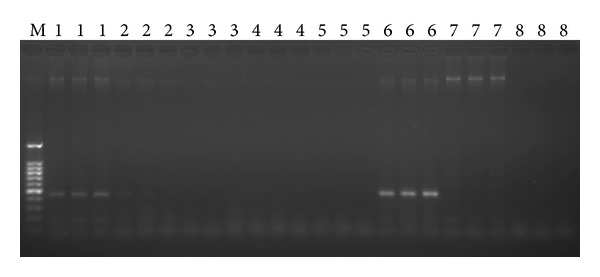
Sensitivity test of conventional PCR based on five-fold serial dilutions of +1 leaf gDNA. M: 100 bp DNA ladder marker; Lane 1, 500 ng/*μ*L; Lane 2, 100 ng/*μ*L; Lane 3, 20 ng/*μ*L; Lane 4, 4 ng/*μ*L; Lane 5, 0.8 ng/*μ*L; Lane 6, positive control; Lane 7, mock control; Lane 8, blank control.

To further validate the sensitivity of this TaqMan method, +1 leaf gDNA of “ROC”22 infected with smut was serially diluted five fold and measured by TaqMan real-time PCR and conventional PCR. The TaqMan assay *R* ^2^ value was 0.998; Ct values ranged from 28.202 ± 0.354 to 36.575 ± 0.330 (Table 1). The minimum detection limit was 0.8 ng/*μ*L with mean Ct value of 36.575 ± 0.330, which was estimated to contain around 41.123 ± 4.953 copies of *bE* target gene (Table 1). When the concentration of the +1 leaf gDNA was reduced to 100 ng/*μ*L, no apparent amplification was observed on agarose gel (Figure 3). These results revealed that the TaqMan assay developed in this study was more sensitive than that of conventional PCR.

### 3.4. Germination Process and Growth Curve of Smut Teliospores

**Figure 4:**
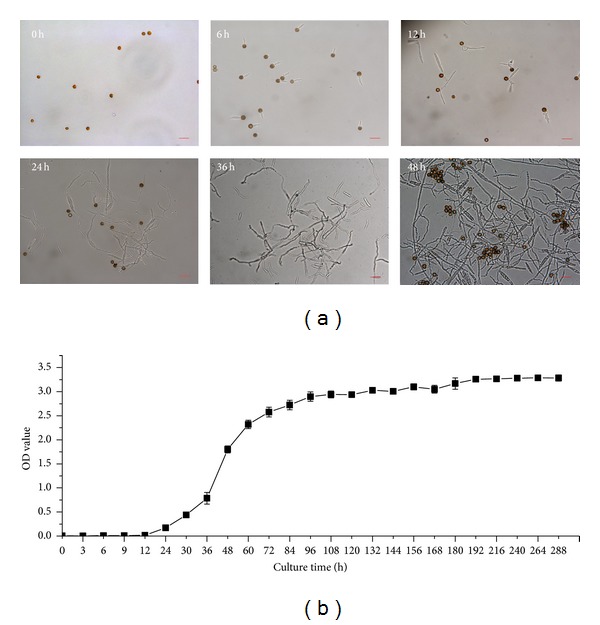
Germination (a) and growth curve (b) of smut teliospores. (a) Teliospore germination monitored by microscopy (magnification ×400), Bar = 20 *μ*m. (b) The growth curve during the culture process (from 0 h to 288 h); all data points mean ± SE (*n* = 3).

The observation of teliospore germination process by microscopy is shown in Figure 4(a). Spores began to germinate after 6 h after inoculation and sprouted considerably at 12 h. The process of spore germination is one germ tube producing one promycelium. The promycelia were found and began to detach at 12 h. There were many free promycelia at 24 h, and these promycelia lengthened as the culturing continued. Some promycelia outgrew basidiospores at 36 h, and a small amount of them began to detach to form microspores. At 48 h, a large number of spores had detached, which lead to many microspores and resulted in turbidity of the liquid medium. In parallel regular samples were measured at OD_600nm_ (Figure 4(b)). A slow phase of smut pathogen growth was observed from 0 h to 12 h; then a logarithmic growth phase appeared from 12 h to 60 h, followed by a stationary phase and declining phase. Based on the above results, the germination process was summarized as follows: teliospore germination and then germ tube, promycelium, basidiospores, and microspores produced one by one. The growth curve could be divided into slow phase (0–12 h), logarithmic growth phase (12–60 h), and stationary phase (60 h-). This will help to understand the correlation between germination and mycelial growth and to further determine the key interaction time of the sugarcane-smut relationship. Therefore, during the following artificially inoculation treatments, 12 h, 24 h, 48 h, 120 h, 168 h, and 336 h were determined to be the sampling time points.

### 3.5. Testing of Smut-Infected Sugarcane Plantlets Using the TaqMan Real-Time PCR Assay

**Figure 5:**
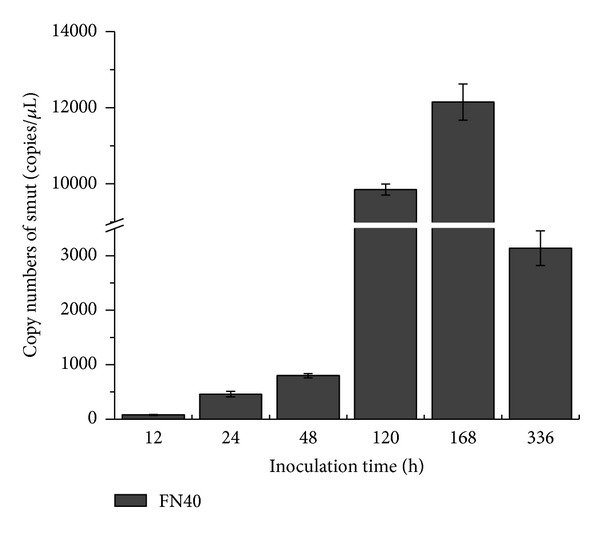
Detection of smut-infected FN40 plantlets. Copy numbers of smut were calculated with the equation of the linear regression line. All data points mean ± SE (*n* = 3).

In order to detect the dynamic range of the assay on infected sugarcane, pathogen-free plantlets of variety FN40 were artificially inoculated with the smut pathogen and genomic DNA extracted from samples at 12 h, 24 h, 48 h, 120 h, 168 h, and 336 h. The sample collected at 0 h was used as the mock control. The standard curve was generated using pbE DNA with the *R* ^2^ value in the linear regression being 0.994. Among these inoculated samples, Ct values ranged from 28.084 ± 0.022 to 35.214 ± 0.034, and the quantity of smut varied from 77.695 ± 6.177 to 12148.273 ± 473.911 (Figure 5). Smut pathogen could be detected within the first 12 h. At 12 h, 77.695 ± 6.177 copies/*μ*L of smut pathogen was detected, and the corresponding fluorescent levels continued to increase from 24 h (456.946 ± 50.077 copies/*μ*L) to 168 h (12148.273 ± 473.911 copies/*μ*L); however, they dropped remarkably at 336 h (3140.044 ± 315.611 copies/*μ*L). It meant, that compared with the copy number of *S. scitamineum* at 12 h in FN40, dynamic 5.9-, 10.3-, 126.8- and 156.4-fold increasing was detected at 24 h, 48 h, 120 h, and 168 h, respectively. The copy numbers decreased by 74.2% at 336 h compared to those at 168 h, but still higher than those of 12 h.

### 3.6. Copy Numbers Assay in Sugarcane Resistant and Susceptible Genotypes Challenged with *S. scitamineum*

**Figure 6:**
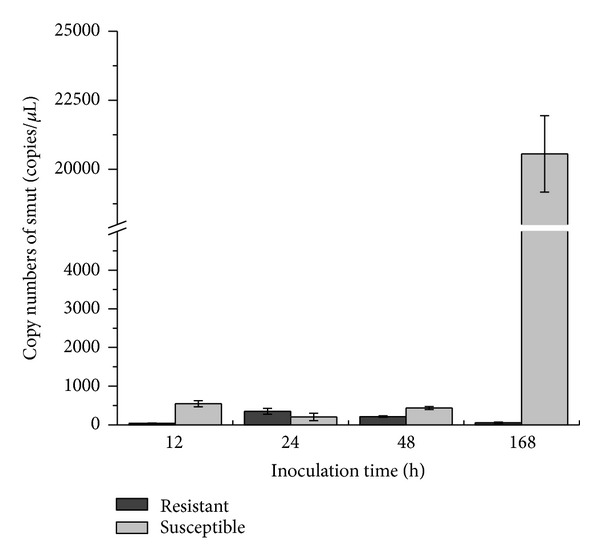
Quantification of the pathogen in sugarcane genotypes challenged with *S. scitamineum*. Resistant: Yacheng05-179; susceptible: “ROC”22. Copy numbers of smut were calculated with the equation of the linear regression line. All data points mean ± SE (*n* = 3).

Sugarcane genotypes Yacheng05-179 (smut resistant) and “ROC”22 (smut susceptible) were artificially inoculated in the buds and used for investigation of the correlation between copy number of *S. scitamineum* and smut resistance. The *R* ^2^ of the standard curve was 0.998 for TaqMan PCR.  Figure 6 showed that the results of the smut pathogen quantification and the difference of copy numbers between resistant and susceptible varieties were significant. It should be noted that no amplification was observed in the mock and blank controls. After challenge, the copy numbers for resistant variety ranged from 43.761 ± 5.464 to 349.772 ± 72.078, with the lowest at 12 h (43.761 ± 5.464) and the highest at 24 h (349.772 ± 72.078) and dropped to 55.569 ± 721.604 at 168 h. In contrast, copies in susceptible variety varied from 205.658 ± 94.320 to 20556.141 ± 1384.162, with much higher conidial densities than those of the resistant variety especially at first sampling point (545.402 ± 80.111) and at last sampling point (20556.141 ± 1384.162), indicating that susceptible variety was more easily infected by the pathogen and more conducive to the pathogen proliferation than the resistant variety.

## 4. Discussion

The detection of smut pathogens is important at the early stages of sugarcane colonization [20] since it is difficult to differentiate it from other fungi based on mycelial morphology. The production of smut sori was observed during 6–12 weeks after pathogen challenge. PCR combined with microscopic examination was used to investigate smut infection in sugarcane during this period, with fungal hyphae beginning to be found 8 weeks after inoculation by microscopy [7]. However, early detection of target pathogen is essential for assessing the health status of plants before the transplanting of plantlets or seedlings into the field [21]. Schenck [22] found that conventional PCR assays were significantly more sensitive and efficient than microscopy for smut pathogen detection. Similarly, Singh et al. [7] found that the smut pathogen could be detected at 12 h after challenge by PCR with primers bE4/bE8, indicating that the PCR assay was more sensitive than that of microscopy. Recently, the specificity of PCR amplification to *bE* gene target of *S. scitamineum *has been validated successfully by several other researchers [3, 7]. Although visible in detecting the presence of the pathogen, conventional PCR was insufficient to quantify the pathogen in nature. Due to its high specificity, sensitivity, accuracy, and speed, real-time PCR is a suitable detection technique [23]. This technique, including TaqMan real-time PCR, has been widely used for the diagnosis of pathogens such as *planta botrytis cinerea* [24], *sugarcane yellow leaf virus* [19],* maize chlorotic mottle virus *[8], and *cucumber vein yellowing virus* [25]. Here, we describe the first report of TaqMan real-time protocol for the sensitive detection and quantification of *S. scitamineum* in sugarcane. The specificity of the TaqMan probe and primers designed according to the sequences of smut *bE* gene was confirmed by TaqMan real-time PCR, and the corresponding PCR product was sequenced and compared to the databases using the BLAST tool. Meanwhile, the specificity of our probe was again determined by searching the nucleotide databases. There was no significant match with any sequence from other organisms. Furthermore, successful detection of smut was achieved in pbE DNA, smut DNA, and sugarcane samples. As there was not any report of closely related genus with smut, the common encountered fungal disease on sugarcane was chosen for detection. Nonpositive result of species-specificity test which was evaluated by DNA samples of *Phoma* sp., *Fusarium moniliforme*,* Pestalotia ginkgo*, *Fusarium oxysporum*, and *Helminthosporium sacchari* and endophytes of Yacheng05-179 and “ROC”22 suggested the good specificity of designed primers and probe for the smut strain and sugarcane.

 With the development of the PCR technique, the required detection sensitivity could be achieved. This method is more convenient than serological technique and hybridization assays using DNA probes, which are time consuming; insensitive and additional technical skills are required. It has been reported that conventional PCR is not suitable for detection of numerous samples by running gels and may even be polluted during post-PCR operation [26], with sensitivity much lower than that of real-time PCR. The main advantages of TaqMan real-time PCR assay are its high sensitivity and reliability (one step and gel free). As reported before, TaqMan real-time PCR method could detect up to 4 fg DNA of *Mycobacterium avium* subsp. *paratuberculosis* [27]. The results obtained in the present study revealed that both the primer pair of bEQ-F/bEQ-R and the TaqMan probe were specific and sensitive to smut pathogen (Figures 1, 2, and 3), and the detection limit for the TaqMan assay was 10 ag (1.987 × 10^1^ copies) of pbE DNA and 10 fg (1.987 × 10^4^ copies) for the conventional PCR, indicating that the sensitivity of the TaqMan assay was 1,000 times than that of conventional PCR (Figures 1 and 2). Further, to evaluate the specificity and sensitivity of the TaqMan assay developed in this study, we also applied it to detect the smut in sugarcane DNA and gained a result with 0.8 ng (41.123 ± 4.953 copies/*μ*L) of +1 leaf gDNA of “ROC”22 infected with smut pathogen, which was 125 times more sensitive than that of conventional PCR (Table 1 and Figure 3).

 Smut spores germinated on the sugarcane internodal surface, and it was followed by the formation of appressoria on the inner scales of the young buds and on the base of the emerging leaves. Entry into the bud meristem occurred between 6 and 36 h after the teliospore deposition [5]. In the present study, we carried out the experiments of spore germination and growth curve observation (Figure 4) in order to establish the correlation between the time of germination and the time of smut mycelial growth, which should lead to a better determination on the key interaction time of sugarcane-smut biosystem. The results indicated that the germination process of *S. scitamineum* was as follows: teliospores germinate, germ tube, promycelium, basidiospore, and microspore, which was similar to the observation of mycelial growth process of *Sporisorium reilianum* in maize seedling by Zhang et al. [28]. The growth curve of smut pathogen could be divided into slow growth phase (0–12 h), logarithmic growth phase (12–60 h), and stationary growth phase (60 h-). Based on the above results, the time points of 12 h, 24 h, 48, 120 h, 168 h, and 336 h after smut pathogen, challenge were chosen as sampling times.

 As more and more sequence data was available for designing primers and probes for specific detection of pathogens, there is no doubt that real-time PCR will become a routine technique in the plant quarantine area [29]. Sugarcane smut is one of the most prevalent diseases affecting sugarcane yield and can cause considerable economic losses [30–32]. As reported, correct quarantine, smut resistant variety releasing, and integrated field management are the three main strategies to control smut disease [3]. Due to a huge amount of spores released by smut whips, it is difficult to stop the infection or reinfection of sugarcane including pathogen-free seedlings or plantlets. Pathogen detection is a crucial procedure in the import and export of sugarcane stalk during germplasm exchange and in the supervision and management of pathogen-free cane or plantlets from tissue culture. However, there is not any report about smut pathogen detection in sugarcane pathogen-free seedlings. In this study, the TaqMan assay was successfully applied to quantify the smut pathogen in tissue cultured plants (FN40) challenged by the pathogen (Figure 5), and the results indicated that this assay was capable to detect the pathogen at the early stage (12 h) of the challenge and at the limit of 456.946 ± 50.077 copies. The new TaqMan real-time PCR technology system can be used to assess whether the sugarcane seed cane, seedlings, or plantlets are really smut pathogen-free. Thus, it is useful in the production and supervision of pathogen-free sugarcane seed cane in the programme of pathogen-free seed cane in mainland China.

 Smut resistance is an important agronomic trait due to the serious loss in sugarcane stalk yield caused by smut pathogen [6, 31, 32]. Based on the TaqMan PCR assay, the copy numbers of the pathogen at 12–168 h in smut resistant variety Yacheng05-179 challenged by *S. scitamineum* were much lower (ranging from 43.761 ± 5.464 to 349.772 ± 72.078) than those of susceptible variety “ROC”22 (ranging from 205.658 ± 94.320 to 20556.141 ± 1384.162) (Figure 6), suggesting the TaqMan PCR assay system developed in this study might be used for smut resistance evaluation if more validation was performed. This TaqMan PCR assay system can be used for evaluation of smut resistance in two, several, or a batch of sugarcane genotypes based on the results achieved. The current method of smut resistance evaluation is still both time and field consuming, using observation of smut whip in at least two sugarcane crops [3, 33–35]. In addition, the sensitive and accurate quantification of the smut pathogen by TaqMan PCR assay is beneficial in giving insight into the mechanisms of sugarcane-smut pathogen interaction.

 In summary, the present study confirmed that these designed primer sets and probe are highly specific and sensitive for smut detection. The TaqMan real-time PCR assay established here can shorten testing time and be used as a tool for the detection and quantification of this pathogen in sugarcane. Its advantages are those as follows: (i) ensuring pathogen-free sugarcane seeds or stalks imported and exported through the sensitive detection of smut pathogen, (ii) providing a new insight into the evaluation of smut resistance of sugarcane genotypes by quantifying the copy numbers of smut pathogen in asymptomatic smut-infected sugarcane, and (iii) gaining supervision and efficient management of pathogen-free sugarcane.

## Figures and Tables

**Table 1:** Mean Ct values and copy numbers obtained by sensitivity test by TaqMan real-time PCR assay.

Concentration of +1 leaf DNA (ng/*μ*L)	Ct values (Ma ± *σ*)	Copy numbers (Ma ± *σ*) (copies/*μ*L)
500	28.202 ± 0.354	12247.062 ± 1579.742
100	30.700 ± 0.391	2246.733 ± 349.973
20	32.895 ± 0.456	509.013 ± 101.842
4	35.561 ± 0.200	81.431 ± 2.540
0.8	36.575 ± 0.330	41.123 ± 4.953

Ma = mean value of three technical replicates; *σ* = standard error.
